# Neodymium-Doped Gadolinium Compounds as Infrared Emitters for Multimodal Imaging

**DOI:** 10.3390/ma16196471

**Published:** 2023-09-29

**Authors:** Maxime Delaey, Seppe Van Bogaert, Ewoud Cosaert, Wout Mommen, Dirk Poelman

**Affiliations:** LumiLab, Department of Solid State Sciences, Ghent University, 9000 Ghent, Belgium; maxime.delaey@ugent.be (M.D.); ewoud.cosaert@ugent.be (E.C.);

**Keywords:** luminescence, near infrared, spectroscopy, neodymium, gadolinium compounds, bioimaging, luminescence decay

## Abstract

This study aims to investigate the optical properties of multiple neodymium-doped gadolinium compounds as a means to examine their eligibility as optical probes for fluorescence imaging. GdVO${}_{4}$, GdPO${}_{4}$, GdAlO${}_{3}$, Gd${}_{2}$SiO${}_{5}$ and Gd${}_{3}$Ga${}_{5}$O${}_{12}$ (GGG) samples were synthesized through solid-state reactions with varying neodymium doping levels to compare their optical properties in great detail. The optimal doping concentration was generally found to be approximately 2%. Furthermore, the luminescence lifetime, which is a valuable parameter for time-gated imaging, was determined to range from 276 down to 14 µs for the highest doping concentrations, resulting from energy transfer and migration assisted decay.

## 1. Introduction

Fluorescence imaging is a biomedical technique in which optical probes are utilized to attain clinically relevant knowledge [1]. Fluorescent dyes and proteins have predominantly been investigated as optical probes; however, inorganic nanoparticles have attracted interest due to their much higher stability, tunable pharmacokinetic properties and resistance to photobleaching [2]. In this work, the possibility of using neodymium-doped gadolinium compounds as optical probes is explored. The presence of gadolinium presents additional advantages: due to its paramagnetic nature and its strong X-ray absorption cross section, the material could also serve as a contrast agent for both magnetic resonance imaging (MRI) and X-ray computed tomography (CT) scans, allowing for true multimodal imaging using a single type of material [3,4].

In order to be an eligible candidate, the material in question should meet various requirements. For the purpose of allowing both in vivo excitation and detection of the emitted light, both the excitation and emission wavelengths of the nanoparticles should lie withing the biological windows, which are wavelength ranges for which the absorption coefficient of biological tissue is minimal. As Nd${}^{3+}$ may be excited at 808 nm and exhibits intense emission around 1060 nm, the relevant biological windows are 650–950 nm and 1000–1350 nm [5]. However the autofluorescence of tissues poses another challenge: removing this background signal from the luminescence of the tissues themselves requires the emission wavelength to be larger than 1100 nm [2] or thee use of time-gated imaging [6]—detecting the particle luminescence after the autofluorescence has decayed—which is made possible through the long decay times of Nd${}^{3+}$ of the order of 100 µs. Lastly, the nanoparticles should be biocompatible and have an appropriate size, as this dictates the pharmacokinetic properties [7].

While in most work, the properties of Nd${}^{3+}$ in only a single host are presented, here, we explore the the luminescence properties of the Nd ions in relation to the structural properties of a series of different hosts. This allows us to pinpoint a number of guidelines in selecting a suitable host for Nd${}^{3+}$-doped near-infrared-emitting phosphors. The optical properties of the bulk materials were investigated in order to examine the influence of the doping concentration and local environment of Nd${}^{3+}$ on the excitation spectra, emission intensities and luminescence lifetimes. This study allows for a founded selection of an optimum material and subsequent development of a suitable nanoparticle synthesis method.

## 2. Materials and Methods

### 2.1. Synthesis

All of the samples were synthesized through solid state reactions with Gd${}_{2}$O${}_{3}$ (99.99%, Alfa Aesar, Ward Hill, MA, USA), Nd${}_{2}$O${}_{3}$ (99.99%, Sigma-Aldrich, Burlington, MA, USA), Al${}_{2}$O${}_{3}$ (99.99%, Alfa Aesar), SiO${}_{2}$ (99.95%, Alfa Aesar), Ga${}_{2}$O${}_{3}$ (99.99%, Sigma-Aldrich), (NH${}_{4}$)${}_{2}$HPO${}_{4}$ (99%, Acros Organics, Geel, Belgium) and V${}_{2}$O${}_{5}$ (99.99%, Thermo Scientific, Waltham, MA, USA) as precursors, which were weighed in a stoichiometric manner such that the chemical reactions listed below are valid. The doping level (*x*) was defined as $\frac{\left[\mathrm{Nd}\right]}{\left[\mathrm{Nd}\right]+\left[\mathrm{Gd}\right]}$. The precursors were mixed using a mortar and pestle, after which they were heated to the temperatures mentioned below, with a heating rate of 300 ${}^{{}^{\circ}}$C/h. The ovens used for synthesis operated in an air atmosphere.

#### 2.1.1. GdAlO${}_{3}$

Gadolinium aluminate was prepared by heating the precursors at 1500 ${}^{{}^{\circ}}$C for 6 h, followed by dry grinding and further heating at 1550 ${}^{{}^{\circ}}$C for 2 h in a tube furnace (ETF 30-50/18-S, Entech, Ängelholm, Sweden).

(1 − *x*)Gd_2_O_3_ + Al_2_O_3_ + *x* Nd_2_O_3_ → 2Gd_1−*x*_Nd_*x*_AlO_3_

#### 2.1.2. Gd${}_{2}$SiO${}_{5}$

Gd${}_{2}$SiO${}_{5}$ was also prepared at 1500 ${}^{{}^{\circ}}$C for 6 h but with the addition of 2 wt% of BaF${}_{2}$ as a flux to facilitate the reaction, as performed in [8]. Afterwards, the samples were heated at 1550 ${}^{{}^{\circ}}$C for 2 h. The same tube furnace was employed to synthesize the batch of Gd${}_{2}$SiO${}_{5}$ samples.

(1 − *x*)Gd_2_O_3_ + SiO_2_ + *x* Nd_2_O_3_ → Gd_2(1−*x*)_Nd_2*x*_SiO_5_

#### 2.1.3. GGG (Gd${}_{3}$Ga${}_{5}$O${}_{12}$)

GGG was prepared at 1450 ${}^{{}^{\circ}}$C for 6 h, followed by dry grinding and further heating at 1500 ${}^{{}^{\circ}}$C for 2 h. Similarly to the last two samples, GGG was also heated in a tube furnace.

3(1 − *x*)Gd_2_O_3_ + 5 Ga_2_O_3_ + 3*x* Nd_2_O_3_ → 2Gd_3(1−*x*)_Nd_3*x*_Ga_5_O_12_

#### 2.1.4. GdPO${}_{4}$

Gadolinium phosphate was synthesized using Li${}_{2}$CO${}_{3}$ (99.998%, Alfa Aesar) as a flux [9]. The precursors and flux were heated to 900 ${}^{{}^{\circ}}$C for 4 h in a muffle furnace (Nabertherm LT 5/13).

(1 − x)Gd_2_O_3_ + 2(NH_4_)_2_HPO_4_ + *x* Nd_2_O_3_ → 2Gd_(1−*x*)_Nd_*x*_PO_4_ + 3H_2_O + 4NH_3_

#### 2.1.5. GdVO${}_{4}$

The precursor mixture was heated up to 800 ${}^{{}^{\circ}}$C for 1 h. Secondly, the precursors were ground and placed an oven at 1100 ${}^{{}^{\circ}}$C for 3 h [4], after which they were heated once more at 1250 ${}^{{}^{\circ}}$C for 2 h. The GdVO${}_{4}$ samples were synthesized in the same muffle furnace that was employed for GdPO${}_{4}$.

(1 − *x*)Gd_2_O_3_ + V_2_O_5_ + *x* Nd_2_O_3_ → 2Gd_(1−*x*)_Nd_*x*_VO_4_

### 2.2. X-ray Diffraction

In order to evaluate the structure of the samples and verify the synthesis process, X-ray powder diffraction (XRD) was performed. The patterns were measured from 5° to 80° with a step size of 0.02° and an integration time of 1.2 s per step for the GGG, GdPO${}_{4}$ and GdVO${}_{4}$ samples, while an integration time of 4.8 s per step of 0.04° for the Gd${}_{2}$SiO${}_{5}$ and GdAlO${}_{3}$ samples was utilized. A θ–2θ diffractometer (Siemens D5000) with Cu Kα radiation (λ = 0.15406 nm) and generator settings of 40 kV and 40 mA was employed.

### 2.3. Scanning Electron Microscopy

Scanning electron microscopy (SEM) was conducted using an FEI Quanta 200 FEG SEM, which operates at high vacuum. Before the morphology of the samples was probed using secondary electrons, the samples were coated with a thin gold layer to prevent them from collecting charges.

### 2.4. Optical Absorption

As the optical absorbance of a powder cannot directly be measured, Kubelka–Munk approximation was employed [10] to convert diffuse reflectance measurements (R(λ)) into absorption:

(1) $$\frac{k(\lambda )}{s(\lambda )}=\frac{{\left(1-R\left(\lambda \right)\right)}^{2}}{2R(\lambda )},$$

where k(λ) is the absorption coefficient, s(λ) is the back-scattering coefficient and R(λ) is the reflectance. The diffuse reflectance measurements were performed on powders that were pressed onto a sample holder and kept into place using a quartz slide. A spectrophotometer (LAMBDA 1050 S UV/Vis/NIR, PerkinElmer, Waltham, MA, USA) equipped with a Spectralon 150 mm integrating sphere with a photomultiplier (PMT) for UV and visible detection and an InGaAs diode for the near-infrared range was then used to obtain the diffuse reflectance spectra of the powders.

### 2.5. Optical Emission

In order to prepare the samples, the powders were pressed onto aluminum sample holders using glass slides to obtain a smooth surface. The slides were removed during the measurements. The emission spectra of the samples were examined with both an Edinburgh FS920 photoluminescence spectrometer using a 450 W Xe arc lamp and a double monochromator as excitation source and a liquid-nitrogen-cooled germanium detector for detection. Spectra were also measured using an InGaAs array spectrometer (AvaSpec- NIR512-1.7-HSC-EVO, Avantes, Apeldoorn, the Netherlands). In the latter setup, the samples were excited with an 808 nm diode laser.

### 2.6. Luminescence Lifetime Measurements

The powders were fixed with carbon tape on an aluminum sheet. The samples were then excited using a Nd:YAG laser-pumped optical parametric oscillator (OPO) tuned to a wavelength of 808 nm and with a pulse repetition rate of 10 Hz (Ekspla NT342, Ekspla, Vilnius, Lithuania). Upon excitation, the samples emitted light, which passed through a 1002 nm long pass filter to remove scattered light produced by the laser from the signal. Similarly, the light resulting from the ${}^{4}$F${}_{3/2}⟶$${}^{4}$I${}_{11/2}$ transition was separated from the rest with the use of filters such that only the luminescence lifetime of that transition was measured. Subsequently, the signal was transferred to an InGaAs amplified photodetector (PDA20C/M, Thorlabs, Newton, NJ, USA) employing an optical fiber (P400-2-VIS-NIR, Ocean Insight, Orlando, FL, USA). Then, the light intensity was transformed into a potential difference in a linear manner. Both the photodetector and trigger of the laser, which served as a start indicator, were connected to a USB oscilloscope (Picoscope 5244D, Pico Technology, St Neots, UK). Given the 3–5 ns pulse duration of the OPO laser and the bandwidth of 5 MHz of the photodetector (rise time of 70 ns), a response time below 100 ns was achieved, which is orders of magnitude lower than the decay time of the Nd${}^{3+}$ luminescence.

## 3. Results

Table 1 encompasses several properties of the researched hosts, namely GdVO${}_{4}$, GdPO${}_{4}$, GdAlO${}_{3}$, Gd${}_{2}$SiO${}_{5}$ and GGG. Upon doping these materials with Nd${}^{3+}$, neodymium ions were substituted on the Gd${}^{3+}$ sites, considering that their ionic radii match closely, as seen in Table 2. Since local symmetry and the environment of these sites vary among the hosts, the host–dopant interactions may differ as well, thus resulting in diverse spectra and luminescence lifetimes. In particular, it is important that the local site symmetry does not contain an inversion center, which causes the transitions to be forced electric dipole transitions instead of electric dipole-forbidden by the mixing of d and f orbitals [11]. Furthermore, the effect of concentration quenching is influenced by the distance between the Nd${}^{3+}$ ions and, as a consequence, the intersite distances in the different hosts.

**Figure 1:**
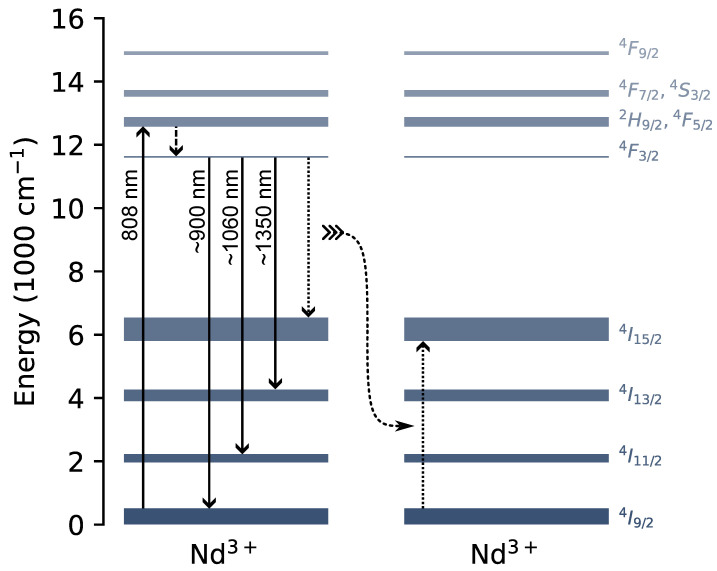
Partial energy diagram of Nd${}^{3+}$ with ground state absorption (upward solid arrow), non-radiative decay (dashed downward arrow), radiative decay (solid downward arrows) and cross relaxation (dotted arrows). Data were taken from [26].

The relevant energy levels and transitions of Nd${}^{3+}$ are shown in Figure 1. Upon excitation by photons with a wavelength of around 808 nm, the system transitions from the ground level into an excited state: ${}^{4}$I${}_{9/2}$⟶${}^{2}$H${}_{9/2}$, ${}^{4}$F${}_{5/2}$. These excited states then decay non-radiatively into ${}^{4}$F${}_{3/2}$. Afterwards, various radiative decay channels towards lower energy states, such as ${}^{4}$I${}_{9/2}$, ${}^{4}$I${}_{11/2}$ and ${}^{4}$I${}_{13/2}$, are possible. These three transitions lead to the main emission features of Nd${}^{3+}$ in the NIR at around 900 nm, 1060 nm and 1350 nm, respectively. Furthermore, in the case of another nearby Nd${}^{3+}$ ion that is in the ground state, cross relaxation may occur as well, in which the excited ion decays to ${}^{4}$I${}_{15/2}$ while exciting the nearby atom to ${}^{4}$I${}_{15/2}$ [21]. Evidently, this phenomenon becomes more prominent with increasing doping levels and contributes to luminescence quenching, as well as migration-assisted decay.

### 3.1. X-ray Diffraction

**Figure 2:**
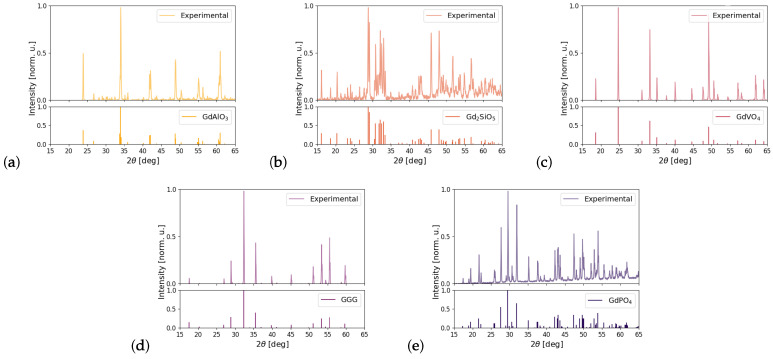
(**a**–**e**) Measured XRD patterns of GdAlO${}_{3}$, Gd${}_{2}$SiO${}_{5}$, GdVO${}_{4}$, GGG and GdPO${}_{4}$ with reference patterns of 00-046-0395, 01-074-1795, 01-086-0996, 01-088-0574 and 00-032-0386, respectively.

GdVO${}_{4}$, GGG and GdPO${}_{4}$ were found to be phase-pure, with the intended stoichiometry and phase and without any traces of impurity phases, while some GdAlO${}_{3}$ samples exhibited peaks that can be attributed to trace amounts of Gd${}_{2}$O${}_{3}$ and some Gd${}_{2}$SiO${}_{5}$ samples that also showed trace amounts of Gd${}_{9.33}$(SiO${}_{4}$)${}_{6}$O${}_{2}$. The XRD patterns did not show any appreciable peak broadening due to finite cystallite size or lattice strain, as seen in Figure 2.

### 3.2. Scanning Electron Microscopy

**Figure 3:**
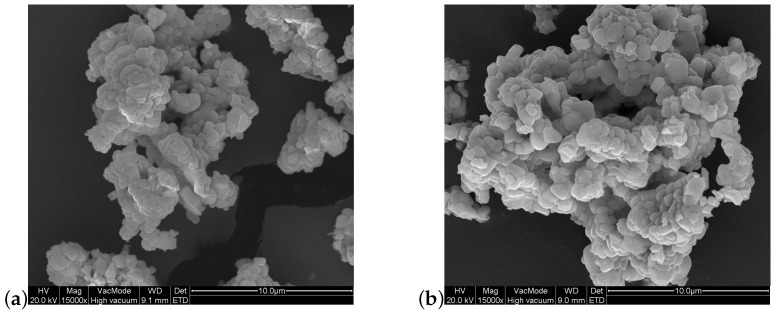
SEM images of (**a**) undoped GdPO${}_{4}$ and (**b**) GdPO${}_{4}$ doped with 2% Nd${}^{3+}$ observed with a magnification of 15,000×.

Figure 3 compares the morphology of doped and undoped GdPO${}_{4}$. As expected, the morphology of the two samples is similar, since the ionic radii of Gd${}^{3+}$ and Nd${}^{3+}$ are comparable. Thus, substituting some gadolinium ions with neodymium ions has little affect on the structure.

**Figure 4:**
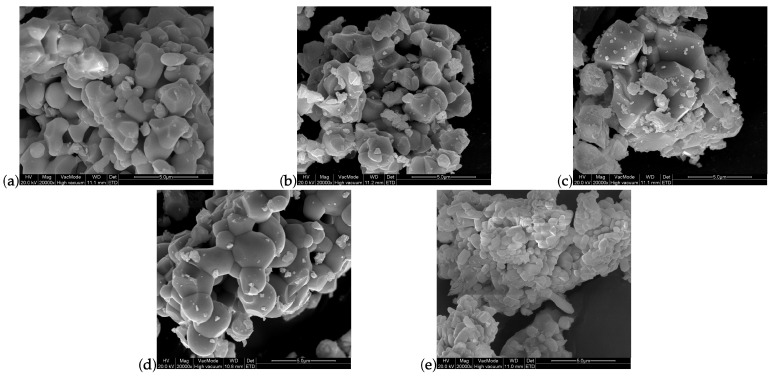
SEM images of (**a**) GdAlO${}_{3}$, (**b**) Gd${}_{2}$SiO${}_{5}$, (**c**) GdVO${}_{4}$, (**d**) GGG and (**e**) GdPO${}_{4}$, all doped with 2% Nd${}^{3+}$ and observed with a magnification of 20,000×.

The SEM images shown in Figure 4 reveal strong agglomeration, as well as similar grain sizes of the order of µm across all samples, with the exception of GdPO${}_{4}$. The latter exhibits smaller structures than the other samples, possibly due to the low heating temperature required for its synthesis.

### 3.3. Optical Absorption

**Figure 5:**
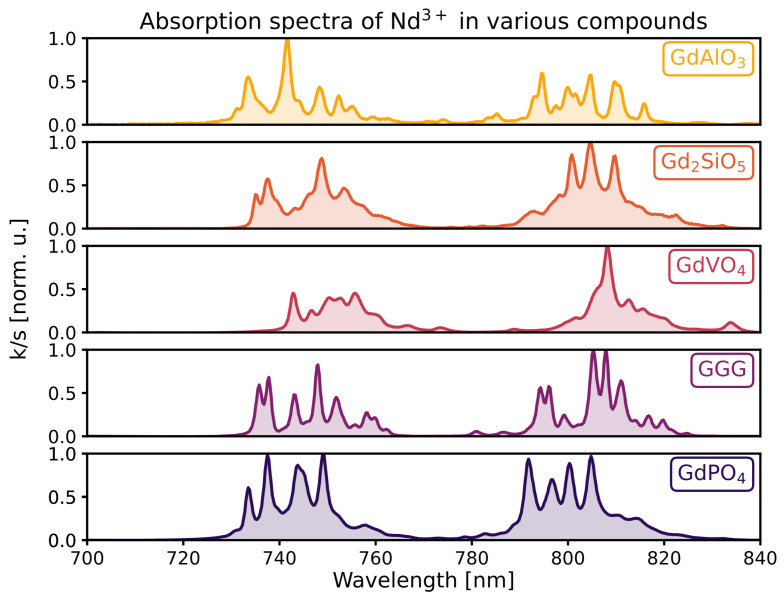
Absorption spectra of the samples doped with 2% neodymium in the region of interest as derived from diffuse reflection spectra.

As can be seen in Figure 5, neodymium-doped GGG and GdVO${}_{4}$ may be appropriately excited within the 730–760 nm and 790–820 nm regions. It has been suggested that excitation of Nd${}^{3+}$ by an 808 nm diode laser could be optimized by tuning its emission wavelength by heating or cooling of the laser [2]. Peculiarly, the absorption cross section of the transitions from ${}^{4}$I${}_{9/2}$ towards ${}^{4}$S${}_{3/2}$ and ${}^{4}$F${}_{7/2}$, roughly around 740 nm, is relatively high in the case of GdPO${}_{4}$ and especially in the case of GdAlO${}_{3}$. These transitions lie within the biological windows as well. One should consider that even within the biological windows, the attenuation coefficient varies. For instance, light with a wavelength of 740 nm is less attenuated by oxygenated whole blood than 800 nm light. However, it is attenuated more by deoxygenated whole blood [5].

**Figure 6:**
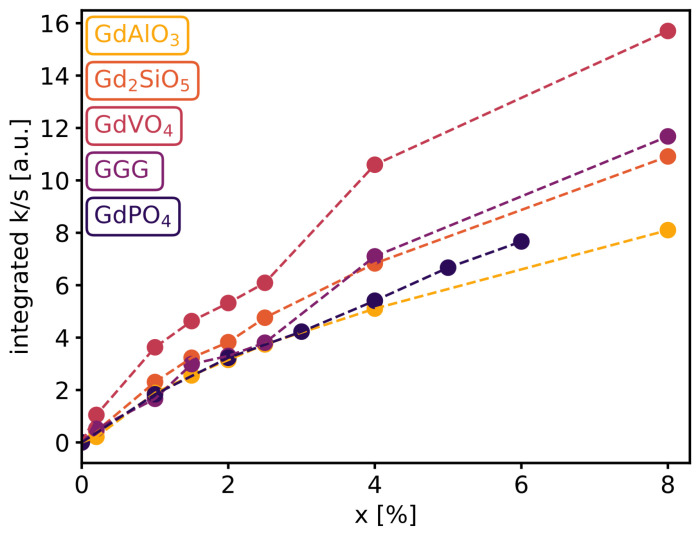
Integrated absorption of the different phosphors as a function of dopant concentration from 720 to 840 nm.

The total integrated absorbance from the transitions seen in Figure 5 obtained by Kubelka–Munk transform is shown in Figure 6 for various doping levels. The trend is similar for all materials; as more neodymium ions occupy gadolinium sites within the host, the absorbance increases. The fact that the absorption increases linearly with dopant concentration for all hosts is a good indication that all Nd is properly incorporated into the host lattice without the occurrence of precipitation or secondary phases.

### 3.4. Optical Emission

**Figure 7:**
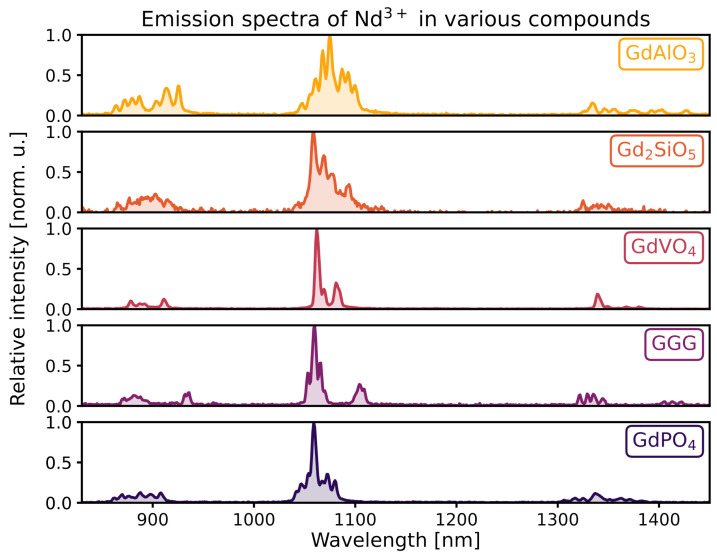
Emission spectra of the samples consisting of various hosts doped with 2% Nd${}^{3+}$.

Figure 7 shows the emission spectra of all samples doped with 2% neodymium. The three transitions discussed in Figure 1 that effectuate radiative decay (${}^{4}$F${}_{3/2}$⟶${}^{4}$I${}_{13/2}$, ${}^{4}$I${}_{11/2}$ and ${}^{4}$I${}_{9/2}$) can be observed among all samples. The branching ratio defines the ratio of the emission intensities of the three main emission peaks.. It is evident that this ratio is most favorable for the 1060 nm peak, corresponding to ${}^{4}$F${}_{3/2}$⟶${}^{4}$I${}_{11/2}$, which is responsible for more than 63% of the emitted intensity in all samples. Remarkably, Nd${}^{3+}$ in GdAlO${}_{3}$ features a maximum shift towards longer wavelengths in contrast to other samples, at 1075 nm. Advantageously, the absorption spectra of many of the most prominent constituents of biological tissue reveal a dip at roughly 1100 nm, and the attenuation decreases from 1060 nm to larger wavelengths until the minimum [5]. As expected from the energy level structure of Gd${}^{3+}$, we did not observe any absorption or emission features related to gadolinium [27]. It is also anticipated that the spectra are similar, as all transitions are between 4f orbitals, which are shielded well from the crystal field by other orbitals. Nonetheless, the local symmetry of the site determines the further splitting of the energy levels. Emission spectra of samples with hosts that have low-symmetry Gd${}^{3+}$ sites appear broader due to the additional transitions. Furthermore, the neodymium ions may be located on two sites with different symmetry in the case of Gd${}_{2}$SiO${}_{5}$.

**Figure 8:**
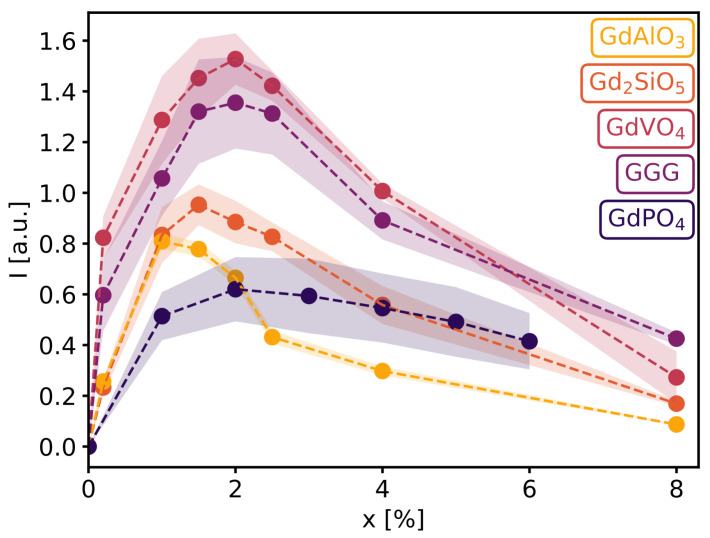
Integrated emission intensity from 1000 to 1200 nm for all samples shown as a function of the doping concentration, with shaded areas indicating the standard error.

However, as seen in Figure 8, showing the integrated emission intensity as a function of dopant concentration, Nd${}^{3+}$ in GdAlO${}_{3}$ is, at most, only half as intense as the brightest sample, which is GdVO${}_{4}$, with a doping level of around 1.5–2.5%; followed by GGG, with 2% Nd${}^{3+}$; Gd${}_{2}$SiO${}_{5}$, with 1.5% Nd${}^{3+}$; GdAlO${}_{3}$, with 1% Nd${}^{3+}$; and, lastly, GdPO${}_{4}$, with 2% Nd${}^{3+}$. These samples exhibit maxima, as an increase in ions results in more available luminescent centers but also amplifies the effect of concentration quenching due to cross relaxation and migration-assisted decay. The maxima of GdAlO${}_{3}$ and Gd${}_{2}$SiO${}_{5}$ appear at lower doping concentrations, which is in line with the distances between Gd${}^{3+}$ sites, as seen in Table 1. Shorter distances between neodymium ions facilitate the transfer of excitation energy from one ion to another, resulting in stronger concentration quenching.

### 3.5. Luminescence Decay

The luminescence lifetime (τ) is an optical property of great significance, especially when considering the application of time-gated imaging. The cross relaxation shown in Figure 1 is a contributor to the concentration quenching of luminescence in neodymium-doped compounds, wherein an ion with an excited state of ${}^{4}$F${}_{3/2}$ partially transfers its energy to a nearby neodymium ion originally in the ground state. Not only does it result in a reduction in emission intensity beyond a certain concentration of Nd${}^{3+}$ ions; it also decreases the luminescence lifetimes. At low doping concentrations, this effect on the decay profiles is well described by [28]:

(2) $$I\left(t\right)=I\left(0\right)e^{-\frac{t}{\tau_{0}}-\Gamma \left(1-\frac{3}{s}\right)\frac{c}{c_{0}}{\left(\frac{t}{\tau_{0}}\right)}^{\frac{3}{s}}},$$

where $\tau_{0}$ is the lifetime for radiative decay in the absence of other nearby Nd${}^{3+}$ ions; *s* = 6, 8 and 10 if the interaction between the ions is dipole–dipole, dipole–quadrupole and quadrupole–quadrupole, respectively; *c* is the dopant concentration; $c_{0}$ a parameter called the critical transfer concentration, defined as $\frac{3}{4\pi R_{0}^{3}}$; and $R_{0}$ is the distance between two ions for which the radiative decay rate is equal to the energy transfer rate. Lastly, Γ is the gamma function. In the case of dipole–dipole interactions, Equation (2) simplifies to:

(3) $$I\left(t\right)=I\left(0\right)e^{-\frac{t}{\tau_{0}}-\sqrt{\pi }\frac{c}{c_{0}}\sqrt{\frac{t}{\tau_{0}}}}.$$

However, at higher concentrations, energy migration effects are inevitable and should be taken into account as well. The excitation energy of a neodymium ion with an energy level of ${}^{4}$F${}_{3/2}$ may migrate to another nearby neodymium ion, resulting in that ion being excited to ${}^{4}$F${}_{3/2}$ [29]. This process may repeat itself, resulting in migration-assisted decay. The rate of migration-assisted decay (*W*) has been described using various method, such as a hopping model or a diffusion model under different assumptions. What most of the results of these methods have in common is a rate that is proportional to the square of the ion concentration in the case of self-quenching, which has also been experimentally observed [30,31]. In the dipole–dipole approximation, the hopping model yields:

(4) $$W=\pi {\left(\frac{2\pi }{3}\right)}^{5/2}c^{2}\sqrt{C_{\mathrm{da}}C_{\mathrm{dd}}},$$

where $C_{\mathrm{da}}$ is a microparameter of the cross relaxation and $C_{\mathrm{dd}}$ is the microparameter of the donor–donor interaction, which corresponds to migration.

Taking both mechanisms into account, the luminescence decay of most neodymium-doped materials in the dipole–dipole approximation is then described by equation [29,30,32]:

(5) $$I\left(t\right)=I\left(0\right)e^{-\frac{t}{\tau_{0}}-\gamma \sqrt{t}-Wt},$$

where both γ and *W* are macroparameters. γ depends linearly on the ion concentration, while *W* is proportional to the square of the ion concentration [29,30,31,32,33]. Therefore, it is possible to write both as:

(6) $$\left\{\begin{array}{c} \gamma =\frac{c}{c_{0}}\sqrt{\frac{\pi }{\tau_{0}}}=c\gamma^{\prime },\\ W=\pi {\left(\frac{2\pi }{3}\right)}^{5/2}c^{2}\sqrt{C_{\mathrm{da}}C_{\mathrm{dd}}}=c^{2}W^{\prime }, \end{array}\right.$$

allowing a sequence of decay profiles derived from samples prepared at a multitude of doping levels to be fit with only three fixed parameters: $\tau_{0}$, $\gamma^{\prime }$ and $W^{\prime }$ (in addition to the amplitude (I(0)) of each sample). The mean lifetime ($\tau_{m}$) can be generally defined according to Equation (7) [28]. By using this definition in conjunction with Equation (5), the mean lifetime can be presented as a function of the doping concentration.

(7) $$\tau_{m}=\frac{\int_{0}^{\infty }tI\left(t\right)dt}{\int_{0}^{\infty }I\left(t\right)dt}$$

**Figure 9:**
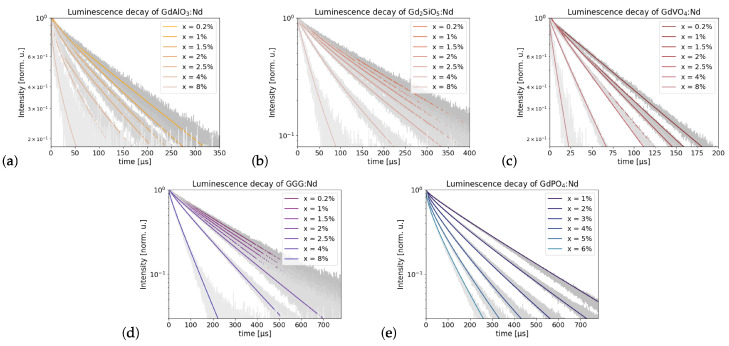
(**a**–**e**) Luminescence decay of GdVO${}_{4}$, Gd${}_{2}$SiO${}_{5}$, GdAlO${}_{3}$, GGG and GdPO${}_{4}$ in semi-log scale respectively, each fit using Equation (5), except (**b**), which was fit using Equation (8).

The decay profiles are shown in Figure 9. Equation (5) was fit to the ensemble of decay profiles for all dopant concentrations simultaneously by minimizing the sum of all the squared errors of each fit using the Levenberg–Marquardt algorithm, while keeping $\tau_{0}$, $\gamma^{\prime }$ and $W^{\prime }$ from Equation (6) fixed across every profile and *c* equal to the nominal neodymium concentration of the synthesis.

It can be seen that this approach properly describes the luminescence decay of a multitude of samples with a limited number of parameters. Small deviations from the experimental data can be attributed to slight deviations of the actual neodymium concentration from the nominal concentration. This process was not conducted for Gd${}_{2}$SiO${}_{5}$, as it has two non-equivalent gadolinium sites in which the Nd${}^{3+}$ may be located, as seen in Table 1. This may also contribute to the non-exponential trend of its luminescence decay, since there are two different environments in which the neodymium ion could reside. Instead, a biexponential fit was performed on each Gd${}_{2}$SiO${}_{5}$ sample separately.

**Figure 10:**
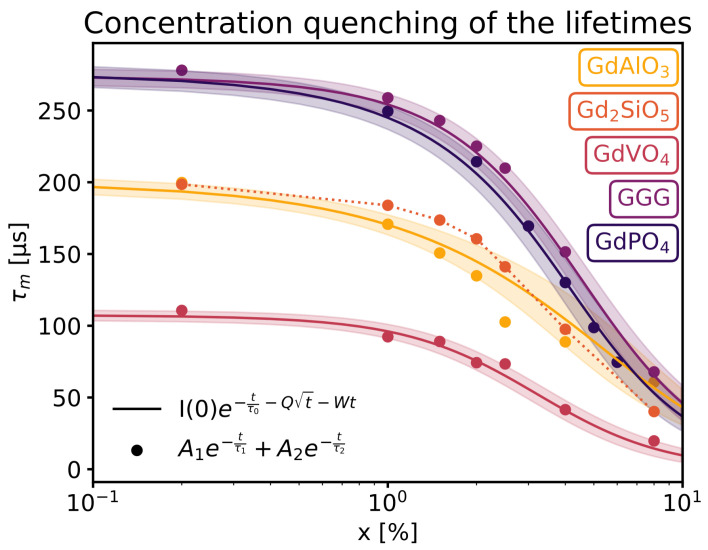
Mean luminescence lifetime as a function of the doping concentration on a semi-log scale, with shaded areas indicating standard error resulting from the errors in the fitting parameters propagated to the lifetimes.

As the used fitting parameters are independent of the dopant concentration, three parameters can be used to construct luminescence decay curves at various doping levels. From these decay curves, the mean lifetime ($\tau_{m}$) can be derived using Equation (7), where the numerator and denominator were numerically calculated. This allows for a continuous visualization of the mean lifetime as a function of the doping level, as seen in Figure 10. To corroborate the validity of this approach, the biexoponential function below (8) was fit on each sample separately, as denoted by dots in Figure 10:

(8) $$I\left(t\right)=A_{1}e^{-\frac{t}{\tau_{1}}}+A_{2}e^{-\frac{t}{\tau_{2}}},$$

with $A_{1}$, $A_{2}$, $\tau_{1}$ and $\tau_{2}$ as parameters. While this results in a lot more parameters with little physical meaning, it does describe the individual decay profiles well. The mean lifetime can once again be calculated using Equation (7). $\tau_{m}$ as a function of doping concentration may be observed in Figure 10, the lifetimes obtained by fitting a biexponential function separately correspond closely to the lifetimes obtained through Equation (5). A comparison with other works was conducted in the case of GdVO${}_{4}$, as shown in Table 3.

From Figure 10, it is also clear that there is a substantial difference in the luminescence lifetime of neodymium doped in a variety of hosts. For instance, the $\tau_{0}$ of neodymium-doped GGG and GdPO${}_{4}$ is nearly three times that of GdVO${}_{4}$. The mean lifetimes of Nd${}^{3+}$ in GdAlO${}_{3}$ and Gd${}_{2}$SiO${}_{5}$ are also similar, with the former having a $\tau_{0}$ of 200 µs.

## 4. Discussion

**Figure 11:**
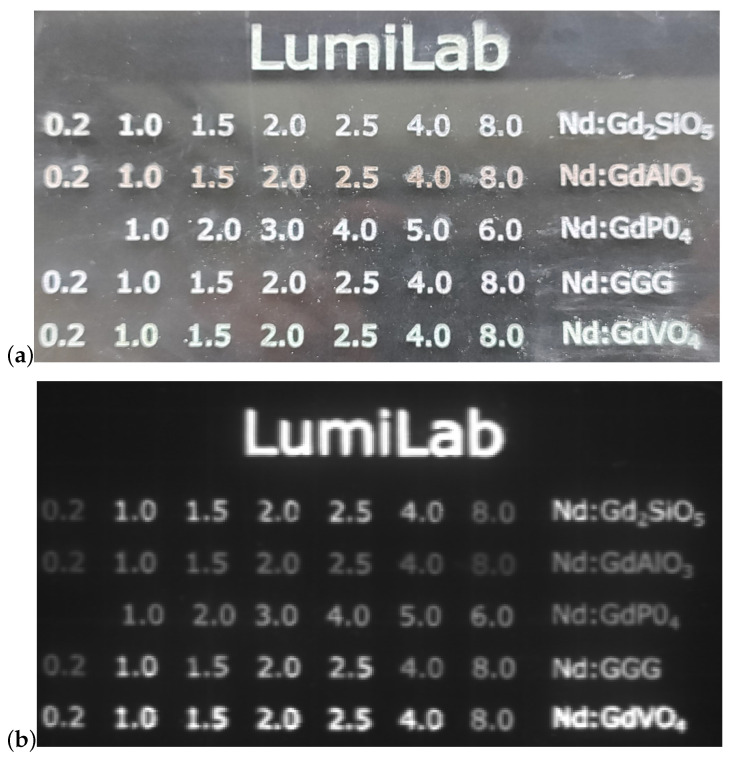
All samples in an acrylic sample holder. Each row corresponds to the host denoted on the right, and the doping concentration (x, in %) is denoted by numbers. Host names comprise the sample with 2% Nd${}^{3+}$, and “LumiLab” consists of GdVO${}_{4}$ doped with 2% Nd${}^{3+}$. (**a**) Sample in visible light and (**b**) illuminated by ambient light photographed in the NIR region with a Xeva 1.7 320 TE3 USB 100 InGaAs camera.

A total of 34 samples with 5 different hosts and varying doping levels were synthesized using solid-state reactions, as shown in Figure 11, which also presents brightness in the NIR.

As seen in Figure 10, the lifetimes vary substantially among the samples, ranging from 276 to 14 µs. Autofluorescence of biological tissues in the near-infrared range is a common source of background signals in bioimaging. Since this autofluorescence has a much shorter decay time—between 0.1 ns and 7 ns [38]—than that of the Nd-doped particles, it is easy to separate the two signals using time-gated imaging. In this study, the autofluorescence signal was suppressed using pulsed excitation, incorporating a delay of at least 10 ns before measuring the signal from the luminescent particles.

The emission intensity is another significant factor. Figure 8 reveals GdVO${}_{4}$ doped with 1.5–2.5% Nd${}^{3+}$ to be the brightest sample. The spectra in hosts with low symmetry gadolinium sites appear broader due to the low symmetry, in the case of Gd${}_{2}$SiO${}_{5}$ further broadening is caused by a second inequivalent site in which the neodymium ions may be located. The width of the peaks separately is a result of the limited resolution of the detectors. This work indicates that the different Nd-doped Gd-based compounds show excellent optical characteristics and are potentially suitable for bioimaging. Ultimately, the choice between the different hosts for bioimaging will come down to the ease of preparation of high-performance nanoparticles with good biocompatibility and a narrow size distribution.

## Figures and Tables

**Table 1:** Structural properties of the hosts.

	GdAlO_3_	Gd_2_SiO_5_	GGG	GdPO_4_	GdVO_4_
Melting point (${}^{{}^{\circ}}$C) [12,13,14,15,16]	~2070	~1950	~1720	1899–1920	~1800
structure [17] 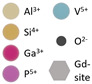	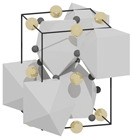	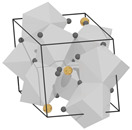	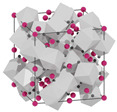	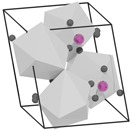	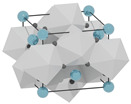
Space group [17]	Pnma	P2${}_{1}$/c	I$\bar{a}$3d	P2${}_{1}$/c	I4${}_{1}$/amd
Gd${}^{3+}$ sites [17,18]	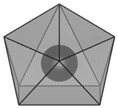	site A: 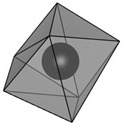 site B: 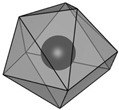	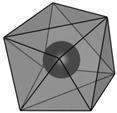	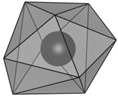	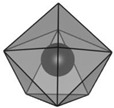
Coordination number [19,20,21,22,23]	8	site A: 7 site B: 9	8	9	8
Local site symmetry [19,20,21,22,23]	C${}_{s}$	site A: C${}_{s}$ site B: C${}_{3v}$	D${}_{2}$	C${}_{1}$	D${}_{2d}$
Distance between Gd${}^{3+}$ sites [Å] [17,24]	2×3.67 2×3.75 2×3.79	$A\left\{\begin{array}{c} 1\times 3.51\\ 2\times 3.57\\ 1\times 3.73 \end{array}\right.$ $B\left\{\begin{array}{c} 1\times 3.36\\ 2\times 3.67\\ 1\times 3.73 \end{array}\right.$	4×3.84 8×5.87 2×6.28	2×4.00 1×4.01 2×4.19	4×3.95 4×5.98 8×6.02

**Table 2:** Ionic radii of Gd${}^{3+}$ and Nd${}^{3+}$ with varying coordination numbers [25].

Coordination Number:	VI	VII	VIII	IX
Ionic radii of Gd${}^{3+}$ [Å]	0.938	1	1.053	1.107
Ionic radii of Nd${}^{3+}$ [Å]	0.983	no data	1.109	1.163

**Table 3:** Luminescent lifetime of Nd${}^{3+}$ in GdVO${}_{4}$.

Doping Level (%)	This Work (µs)	Other Work (µs)	Reference
0.5	104	107, 88	[4,34]
0.9	98	97	[32]
1	96	95, 88, 84	[4,35]
1.2	92	90	[36]
2	75	81–63, 44	[4,37]
5	31	34, 26	[4,37]
10	10	9	[4]

## Data Availability

Data available upon reasonable request from the authors.
